# Bisphenol A Does Not Mimic Estrogen in the Promotion of the In Vitro Response of Murine Dendritic Cells to Toll-Like Receptor Ligands

**DOI:** 10.1155/2017/2034348

**Published:** 2017-07-25

**Authors:** Marita Chakhtoura, Uma Sriram, Michelle Heayn, Joshua Wonsidler, Christopher Doyle, Joudy-Ann Dinnall, Stefania Gallucci, Rebecca A. Roberts

**Affiliations:** ^1^Laboratory of Dendritic Cell Biology, Department of Microbiology-Immunology, Lewis Katz School of Medicine, Temple University, Philadelphia, PA 19140, USA; ^2^Division of Rheumatology, Joseph Jr. Stokes Research Institute, The Children's Hospital of Philadelphia, Philadelphia, PA 19104, USA; ^3^Department of Biology, Ursinus College, Collegeville, PA 19426, USA; ^4^Biochemistry and Molecular Biology Program, Ursinus College, Collegeville, PA 19426, USA

## Abstract

Sex hormones affect immune responses and might promote autoimmunity. Endocrine disrupting chemicals such as bisphenol A (BPA) may mimic their immune effects. Conventional dendritic cells (cDCs) are pivotal initiators of immune responses upon activation by danger signals coming from pathogens or distressed tissues through triggering of the Toll-like receptors (TLRs). We generated in vitro murine cDCs in the absence of estrogens and measured the effects of exogenously added estrogen or BPA on their differentiation and activation by the TLR ligands LPS and CpG. Estrogen enhanced the differentiation of GM-CSF-dependent cDCs from bone marrow precursors in vitro, and the selective estrogen receptor modulators (SERMs) tamoxifen and fulvestrant blocked these effects. Moreover, estrogen augmented the upregulation of costimulatory molecules and proinflammatory cytokines (IL-12p70 and TNF*α*) upon stimulation by TLR9 ligand CpG, while the response to LPS was less estrogen-dependent. These effects are partially explained by an estrogen-dependent regulation of TLR9 expression. BPA did not promote cDC differentiation nor activation upon TLR stimulation. Our results suggest that estrogen promotes immune responses by increasing DC activation, with a preferential effect on TLR9 over TLR4 stimulation, and highlight the influence of estrogens in DC cultures, while BPA does not mimic estrogen in the DC functions that we tested.

## 1. Introduction

Conventional dendritic cells (cDCs) are pivotal regulators of the immune system that initiate immunity or immunological tolerance depending on their state of activation [1, 2]. cDCs activate upon exposure to danger signals coming from pathogens [3] or distressed tissues [4, 5], which trigger pattern recognition receptors like the Toll-like receptors (TLRs) [6]. Upon TLR stimulation, cDCs upregulate costimulatory molecules and proinflammatory cytokines to stimulate T lymphocytes and initiate immune responses [7, 8]. Many of the studies that have analyzed the activation of cDCs were conducted using the in vitro model of cDCs generated from bone marrow precursors in the presence of the growth factor GM-CSF [9, 10]. These cDCs express TLR4, which is triggered by the Gram-negative bacteria-derived lipopolysaccharide (LPS) or by the cytokine HMGB1 [11, 12], and also TLR9, which is stimulated by CpG oligonucleotide sequences that are exposed by viruses or necrotic cells [13]. Therefore, TLRs are important stimulators of cDCs in host defense, in autoimmunity [14], and in transplant rejection [15].

The environment in which DCs differentiate strongly affects their ability to polarize immune responses [16] and the role of sex hormones in DC biology requires further investigation. Evidence suggests that sex hormones influence the immune response [17] and promote some autoimmune diseases [18]. This pathogenic link becomes more worrisome when we consider the increased introduction of estrogens in the food chain through administration of hormones to animal stock and the presence of environmental endocrine disrupting chemicals, such as bisphenol A (BPA), which can act as xenoestrogens [19].

BPA is used in the manufacture of polycarbonate, epoxy, and polyester styrene resins. There are many routes of exposure to this endocrine disruptor, as BPA is used in food packaging. Although it has been eliminated from many plastic products for infants, it is still used in can linings and is also found in household dust and drinking water [20–27]. Predominantly, BPA enters the body through the digestive tract [22], where it can bind estrogen receptors or act through nonclassical pathways. BPA has been proposed to affect the immune system, but more investigations are necessary to support this hypothesis [19].

17beta-estradiol (E2), the most common circulating form of estrogen, in complex with its intracellular receptors, acts as a transcription factor that regulates gene expression [16]. cDCs, including cDCs grown in GM-CSF, express estrogen receptors [28, 29] and E2 increases migration and activation of human DCs [30]. In mouse DCs, E2 was reported to promote cDC differentiation and survival in vitro and increase their expression of costimulatory molecules upon exposure to TLR ligands LPS, CpG, or Poly I:C [31–34]. The role of estrogens in cDC production of proinflammatory cytokines, which are pivotal mediators of the cDC stimulatory activity, remains controversial. GM-CSF cDCs generated in culture in the absence of estrogens showed a reduced production of IL-12 upon exposure to LPS or CpG [34]. However, GM-CSF cDCs generated in culture from estrogen receptor alpha- (ERalpha-) deficient mice produced larger amounts of IL-12 with LPS and slightly decreased amounts of IL-12 and TNF*α* cytokines with CpGs, suggesting that estrogens may have different effects on DC response to individual TLRs [29]. DCs from lupus-prone mice that are deficient for ERalpha produced decreased amounts of IL-6 upon TLR stimulation [35]. Therefore, it remains important to investigate the impact of estrogens on cDC differentiation and activation.

Whether BPA activates or suppresses immune responses and autoimmunity needs clarification [19, 36–40]. In cDCs, BPA at high concentrations either promoted [41] or reduced DC differentiation [42] and did not have effects on DC activation [42], while the effects of BPA at concentrations comparable to those present in the human body are still unknown [43].

We developed a protocol to generate cDCs in the absence of estrogens. With this new tool, we studied the effects of estrogens and BPA on the response of DCs to proinflammatory TLR stimulation. Our data show that estrogen enhances cDC differentiation in the presence of GM-CSF, and their activation upon TLR stimulation, partially via increasing TLR expression. Using BPA concentrations that are compatible with in vivo exposures, we found that BPA does not mimic the proinflammatory effects of estrogen, and therefore, its immunomodulatory effects, if any, may require synergisms with other immune modulators.

## 2. Material and Methods

### 2.1. Mice

C57BL/6 mice (Jackson Laboratory) were bred and maintained in our colonies at the Children's Hospital of Philadelphia and at Temple University, which are both American Association for the Accreditation of Laboratory Animal Care-accredited facilities, in accordance with the guidelines of the Institutional Animal Care and Use Committees of both institutions.

### 2.2. Isolation and Culture of Bone Marrow-Derived DCs

Bone marrow was flushed from the femurs and tibiae of 6–12-week-old female C57BL/6 mice using cold PRFCT IF-10 (phenol red-free Iscove's Modified Dulbecco Medium (IMDM) supplemented with 10% charcoal-treated heat-inactivated fetal bovine serum (HyClone), 0.5 mM L-glutamine, penicillin (100 units/mL), streptomycin (100 units/mL), 50 mg/mL gentamycin, and 0.1% beta-mercaptoethanol). T and B lymphocytes were depleted with anti-B220 and anti-Thy1.2 microbeads followed by removal through LS columns (Miltenyi Biotec) according to manufacturer instructions. The depleted population was washed and resuspended in PRFCT DC medium (PRFCT IF-10 supplemented with 3.3 ng/mL GM-CSF) and plated at a density of 10^6^ cells/mL/well in 24-well Costar flat-bottom plates. 50 mM *β*-estradiol 3-benzoate in acetone and 50 mM bisphenol A in methanol were serially diluted with PRFCT IF-10 prior to addition to DC cultures such that the final concentration of acetone or methanol was <0.001%. On days 2 and 5, 1 mL of medium was removed and replaced with 1 mL of PRFCT DC medium and, where appropriate, fresh E2 or BPA were added.

Alternatively, bone marrow precursors were plated without T and B cell depletion in a standard medium (IMDM complete medium supplemented with 10% heat-inactivated fetal bovine serum, 0.5 mM L-glutamine, penicillin (100 units/mL), streptomycin (100 units/mL), 50 mg/mL gentamycin, and 0.1% beta-mercaptoethanol) supplemented with fulvestrant (1 *μ*M or 100 nM in DMSO) and tamoxifen (10 nM or 100 nM in ethanol) (Sigma-Aldrich). 1 mL of media was added on day 2, and 1 mL of media was replaced on day 5 and each subsequent day until the stimulation of the cells.

Resting cDCs were stimulated on day 6 with either 100 ng/mL LPS or 10 *μ*g/mL CpG-B 1826 and harvested after 24 h for flow cytometric analysis, ELISA analysis, and qRT-PCR.

### 2.3. Cytokine Production

Cytokine production (IL-12p70 and TNF*α*) was measured in cell culture supernatants that were stored at −20°C prior to analysis, using ELISA kits (BD Pharmingen and R&D Biosystems).

### 2.4. Flow Cytometry

DCs were harvested on days 6-7 in cold PBS by vigorous pipetting. Following manual counting by trypan blue exclusion, cells were washed and incubated for 20 minutes on ice with Fc*γ*R Block (2.4G2 clone) and for 30 minutes with the following antibodies: APC-conjugated hamster anti-mouse CD11c, PE-CD80, −CD86, −CD11b, FITC-hamster anti-mouse CD40, PerCPCy5.5-anti-MHC class II, and PeCy7-anti-Gr-1, which stains Ly6C and Ly6G. Cells were washed, fixed in 1% formalin, and analyzed on a FACS Calibur/FACS Canto flow cytometer (BD Biosciences) using CellQuestPro/Flowjo softwares.

### 2.5. Quantitative RT-PCR

Gene expression in cDCs was analyzed by real-time quantitative reverse transcriptase-PCR (qRT-PCR) using TaqMan probes as described before [44, 45]. Briefly, RNA was extracted using Qiagen RNasy plus kit (Qiagen Inc., Valencia, CA, USA), following the manufacturer's protocols. cDNA was synthesized using the cDNA archive kit (Life Technologies, Grand Island, NY, USA). TaqMan primers and probes for *TLR4*, *TLR9*, and *ERalpha* were purchased from Applied Biosystem. Cyclophilin was used as the reference gene for normalization. The Ct method of relative quantification of gene expression was used for these TaqMan PCRs (ΔΔCt), and the normalized Ct values (against cyclophilin) were calibrated against the control sample in each experiment.

### 2.6. Statistical Analysis

Data was analyzed using Prism software (GraphPad, San Diego) and ANOVA and post hoc multiple comparisons against the control. Values of *p* < 0.05 (marked in the figures as ^∗^*p* < 0.05, ^∗∗^*p* < 0.01, and ^∗∗∗^*p* < 0.001) were considered significant.

## 3. Results

### 3.1. Estrogen Increases the In Vitro Differentiation of Dendritic Cells

**Figure 1:**
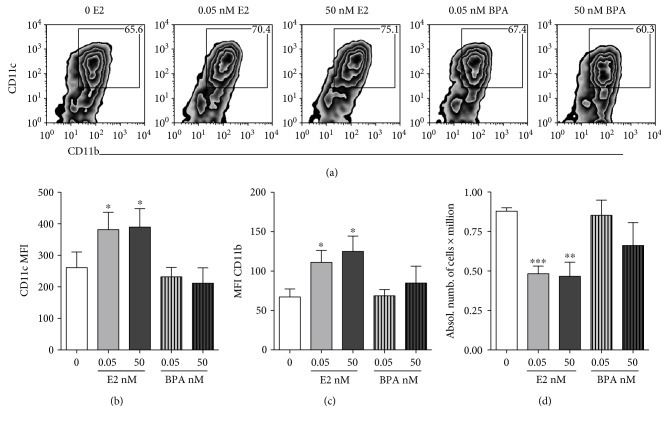
Estrogen enhances the differentiation of cDCs while BPA does not affect cDCs. (a–d) Days 6-7 of culture of cDCs grown in the absence or presence of low (0.05 nM) and high doses (50 nM) of 17beta-estradiol (E2) or low (0.05 nM) and high doses (50 nM) of BPA. (a) Representative plot of the flow cytometry analysis of the cDC differentiation markers CD11c and CD11b expression in the alive gate of cDCs. (b) MFI of CD11c expression. (c) MFI of CD11b expression, at days 6-7 of culture. (d) Results of manual cell counts of cells alive in the same DC cultures in which dead cells positive for the trypan blue staining were excluded; the results are averages and SE of four experiments conducted with four independent BMDC cultures. ∗ represents *p* < 0.05, ∗∗ for *p* < 0.01, and ∗∗∗ for *p* < 0.001.

To investigate the role of estrogen in cDC functions, we cultured murine bone marrow (BM) precursors in the presence or absence of estrogen. It has been previously reported that in the absence of estrogens, the differentiation of cDCs from BM precursors elicited by GM-CSF, using the medium RPMI, was very poor [31]. Therefore, we used a richer medium, Iscove's Modified Dulbecco Medium (IMDM), that is recommended for highly demanding cultures. We analyzed the differentiation of cDCs from BM precursors grown in GM-CSF-enriched complete phenol red-free IMDM lacking estrogens and containing charcoal-treated fetal bovine serum, which is depleted of all steroidal hormones, including estrogens. In some wells, we added 0.05 nM of 17beta-estradiol (E2), a concentration of E2 in the range of what is present in the normal FBS and in the serum of female mice in diestrus (0.05–0.1 nM) [16]. In other wells, we added 50 nM E2, which is comparable to the E2 levels detectable during pregnancy [16]. At days 6-7 of culture, a time when cDCs have completed their differentiation, we found that estrogen is not absolutely necessary for cDC differentiation, but it augments cDC differentiation. As shown in Figure 1(a), cells grown in the absence of steroid/sex hormones have a lower percentage of CD11c-CD11b double-positive cells than cells grown in the same medium supplemented with E2. Moreover, we found that in comparison with cells grown in the absence of steroid/sex hormones, the supplementation with E2 significantly increased the mean fluorescence intensities (MFI) of both CD11c and CD11b surface markers (Figures 1(b) and 1(c)), suggesting that, although some cDCs can differentiate in the absence of E2, this hormone increases the percentages of cDCs and upregulates the expression of differentiation markers. E2 also decreased the absolute numbers of cells in the culture (Figure 1(d)), an effect that is likely due to promotion of cell differentiation and possibly inhibition of cell division by E2, rather than induction of cell death. In order to directly test whether estrogen affects cDC survival, we measured the percentage of cDCs that survived upon stimulation with 100 ng/mL of LPS, a treatment that induces strong DC activation but also can reduce DC survival [46]. We observed that in our culture conditions, estrogen protected cDCs from LPS-induced cell death (Supplemental Figure 1 available online at https://doi.org/10.1155/2017/2034348), indicating that estrogen does not kill cDCs but rather promotes their differentiation and survival.

Since it was previously reported that generating DCs from bone marrow precursors in the presence of GM-CSF yields a mixed population [47] with two main subsets, one of E2-dependent CD11c + CD11b^int^ Ly6C-negative cells and the other of E2-independent CD11c + CD11b^hi^ Ly6C+ cells [32], we determined the presence of these two subsets in our culture. We used the anti-Gr-1 Ab that binds both Ly6C+ and Ly6G+ positive cells and found that our protocol yields a majority (80%) of CD11c + CD11b + Ly6C/G-negative cells and only a minority (10%) of CD11c + CD11b^hi^ Ly6C/G+ cells. The latter cells were neither selected nor inhibited in their development by estrogen (Supplemental Figure 2A), indicating that our protocol yields cDCs in which the effects of estrogens are not confounded by the selection of a different innate subset.

### 3.2. BPA Does Not Promote the In Vitro Differentiation of cDCs

To determine the effects of BPA on cDC differentiation, we cultured bone marrow precursors in the absence of estrogens (as described above), with or without the addition of the E2 analogue BPA. Total urinary BPA levels in humans have been detected in the range of 0–640 nM, with a mean of 11 nM in Americans over the age of six [43]. Therefore, we tested 0.05 nM and 50 nM of BPA, in analogy with the doses of E2 used in the same experiments, and in the range of the concentrations found in the American population. Our results show that even in cells grown in the higher-than-mean concentration of BPA (50 nM), the percentages of expression and the MFI of cDC differentiation markers CD11c and CD11b were comparable to those present in cells generated without E2, suggesting that BPA does not affect cDC differentiation (Figures 1(a), 1(b), and 1(c)). In discordance with what was previously reported [19, 41], BPA did not inhibit the proliferation of bone marrow precursors, since it did not affect the absolute numbers of cDCs (Figure 1(d)) and did not protect them from LPS-induced cell death (Supplemental Figure 1). These findings indicate that at doses that can be physiologically present in the body, BPA does not have E2-like promoting effects on the differentiation and survival of cDCs.

### 3.3. SERMs Decrease the In Vitro Differentiation of Dendritic Cells

**Figure 2:**
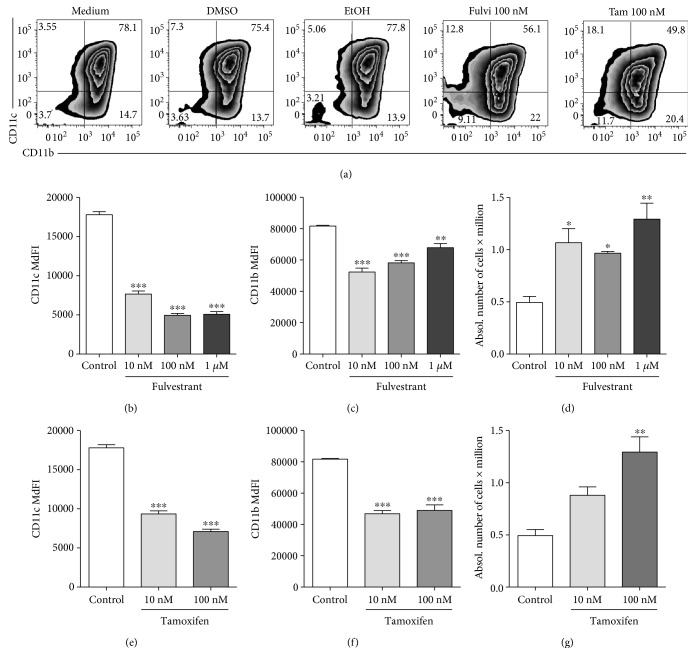
SERMs decrease the in vitro differentiation of dendritic cells. Days 6-7 of the culture of cDCs grown in the regular medium and in the absence or presence of fulvestrant or tamoxifen. (a) Representative plot of the flow cytometry analysis of CD11c and CD11b expression in the alive gate of cDCs generated in standard medium supplemented with 100 nM fulvestrant or 100 nM tamoxifen. (b–g) MdFI of CD11c (b, e); CD11b (c, f); manual counts of alive cells (d, g). Results are averages and SE of biological triplicates conducted with one independent cDC culture. Statistical significance was calculated by one-way ANOVA and post hoc multiple comparison test against the 0 condition (no E2 nor BPA) in a-0063 and against control in (d–i). ∗ represents *p* < 0.05, ∗∗ for *p* < 0.01, ∗∗∗ for *p* < 0.001.

To support the results obtained with cDCs generated in hormone-depleted conditions and supplemented with E2, we studied cDC differentiation in standard medium supplemented with the selective estrogen receptor modulators (SERMs) tamoxifen and fulvestrant. These compounds block E2 from binding to its receptors and ablate E2 signaling, while all other metabolites, which are otherwise removed by charcoal treatment, remain in the culture. We found that the SERMs fulvestrant and tamoxifen decreased the percentage of differentiated cDCs (Figure 2(a)). The expression of the differentiation markers CD11c and CD11b were decreased in the presence of the SERMs fulvestrant and tamoxifen in a dose-dependent manner (Figures 2(b), 2(c), 2(e), and 2(f)). Moreover, we found that the yield of absolute cell numbers increased in the presence of the SERMs (Figures 2(d) and 2(g)). We propose that estrogens accelerate cDC differentiation, and therefore diminish precursor proliferation. Furthermore, SERMs neither selected nor inhibited the differentiation of CD11c + CD11b^hi^ Ly6C/G+ cells (Supplemental Figure 2B), confirming that with the use of our protocol, estrogens do not select for specific innate subsets. All together, these results mirror the effects of the absence of estrogens (Figures 1(a), 1(b), 1(c), and 1(d)). Therefore, we show with two complementary approaches that IMDM promotes cDC differentiation in the absence of estrogens or estrogen signaling and that estrogens have the capability to further enhance cDC differentiation.

### 3.4. Estrogen and BPA Do Not Affect cDC Production of Proinflammatory Cytokines upon Stimulation with the TLR4 Ligand LPS

**Figure 3:**
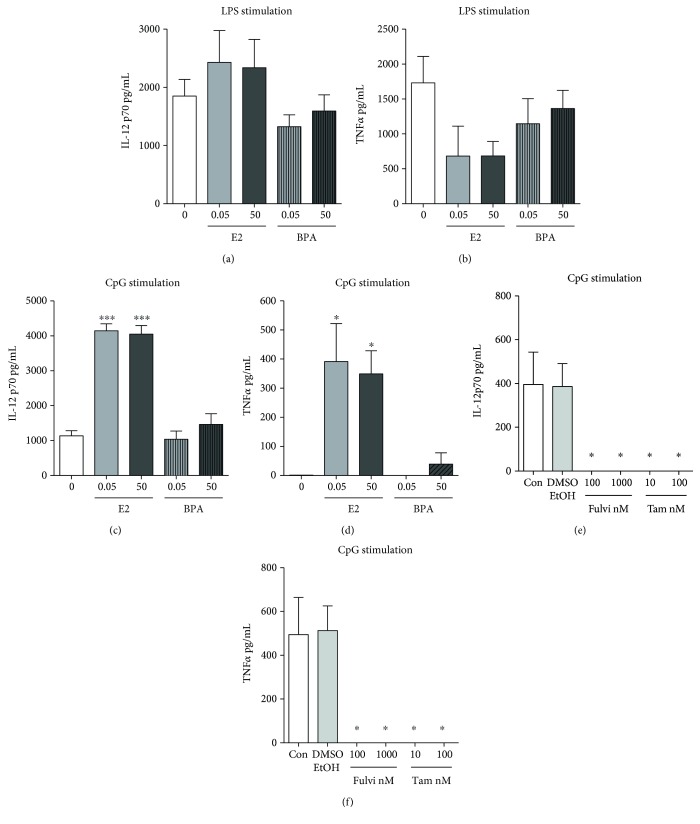
Estrogen but not BPA regulates the production of proinflammatory cytokines by cDCs upon TLR stimulation. We grew cDCs in the absence or presence of low (0.05 nM) and high doses (50 nM) of E2 or BPA for 6-7 days (a–d) or in regular medium in the presence or absence of SERMs fulvestrant or tamoxifen (e-f). We then stimulated cDCs with either 100 ng/mL of LPS (a-b) or 10 *μ*g/mL of CpG (c–f) and measured IL-12p70 and TNF*α* levels by ELISA in DC culture supernatants 24 hours post stimulation. Results are the averages and SE of four DC cultures (a-b, c-d) and two DC cultures with two biological duplicates each (e-f). Statistical significance was calculated by one-way ANOVA and post hoc multiple comparison test against 0 condition (no E2 nor BPA) or control. DMSO and EtOH were used as vehicle controls. ∗ represents *p* < 0.05, ∗∗ for *p* < 0.01, and ∗∗∗ for *p* < 0.001.

It has been reported that cDCs grown in steroidal hormone-depleted medium are deficient in their response to the TLR4 ligand LPS [32, 34]. Since our culture conditions allow the cDCs to differentiate in the absence of E2, we investigated the response of cDCs to LPS in our system. We first measured the production of proinflammatory cytokines. In the absence of any stimulus, cDCs produced undetectable levels of IL-12p70 and TNF*α* regardless of the presence of E2 (data not shown), indicating that we are generating truly resting DCs with no proinflammatory activity. Upon LPS stimulation, cDCs generated in the absence of E2 produced high levels of IL-12p70 and TNF*α* (Figures 3(a) and 3(b)). In particular, cDCs generated in the absence of E2 produced an amount of IL-12 that was not significantly different from that produced by cDCs generated with diestrus levels of E2 (Figure 3(a)). High levels of E2 (50 nM) did not increase or decrease such IL-12 levels. cDCs generated in the presence of the endocrine disruptor BPA produced slightly less IL-12p70 than cDCs grown in the absence of E2, but the differences were small and not statistically significant (Figure 3(a)). BPA also did not significantly affect the production of TNF*α* by cDCs, and cDCs generated in the presence of E2 produced lower, though not significantly different, levels of TNF*α* (Figure 3(b)). These results indicate that E2 and BPA do not influence the production of proinflammatory cytokines upon TLR4 stimulation of cDCs.

### 3.5. Estrogen but Not BPA Augments CpG-Induced Production of Inflammatory Cytokines

We studied the effect of E2 on the induction of cytokines in cDCs by CpG and found that E2 has a major impact on the response of cDCs to CpG (Figures 3(c) and 3(d)). Indeed, cDCs generated in the absence of E2 produced moderate levels of IL-12p70 and undetectable levels of TNF*α*, while those generated in diestrus levels of E2 responded to CpG by producing significantly higher amounts of IL-12p70 and TNF*α*, similar to those that we see in cDCs generated in the presence of a standard fetal bovine serum containing physiological levels of hormones [44, 45]. These results suggest that the optimal induction of proinflammatory cytokines by TLR9 stimulation occurs only in cDCs differentiated in the presence of E2. Increasing the amount of E2 did not further increase the production of proinflammatory cytokines upon CpG stimulation (Figures 3(c) and 3(d)). The poor response to CpG, or complete lack of response to CpG, as in the case of TNF*α* production, in the absence of E2 was not rescued by BPA, irrespective of the concentration we used. This indicates that this E2 analogue cannot substitute for the physiological hormone in the process of differentiation or activation that makes DCs capable of fully responding to TLR9 ligands (Figures 3(c) and 3(d)). Moreover, we found that SERMs fulvestrant and tamoxifen inhibited the production of both cytokines induced by CpG stimulation in standard conditions, confirming that the cytokine response to TLR9 stimulation is enhanced by estrogens (Figures 3(e) and 3(f)).

### 3.6. Estrogen but Not BPA Promotes TLR-Induced Expression of MHC Class II and Costimulatory Molecules

**Figure 4:**
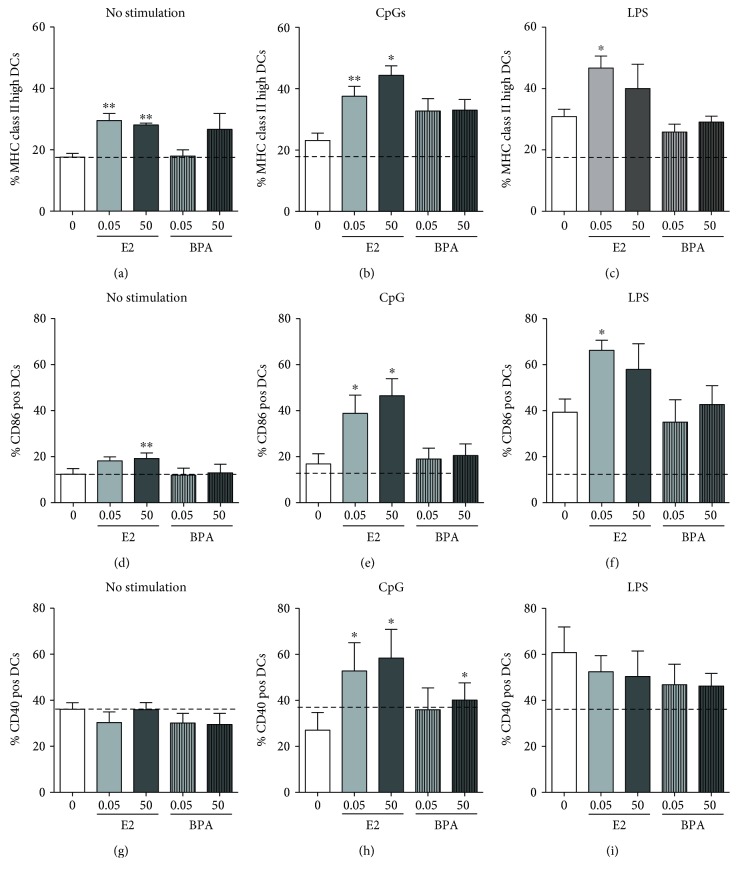
Estrogen but not BPA upregulates the expression of costimulatory molecules upon stimulation with CpG and LPS. (a–i) We grew cDCs in the absence or presence of low (0.05 nM) and high doses (50 nM) of E2 or BPA for 6-7 days. We then stimulated cDCs with either 100 ng/mL of LPS (c; f; i) or 10 *μ*g/mL of CpG (b; e; h) as described in Figure 2 and analyzed the percentages of cDCs (gated for CD11c+ cells) positive for the indicated costimulatory and MHC molecules, 24 hours post stimulation with TLR ligands. Results are averages and SE of four BMDC cultures. Dotted lines indicate baseline expression in unstimulated cDCs grown in the absence of E2 or BPA; pos indicates positive. For each costimulatory or MHC molecule, we calculated statistical significance by one-way ANOVA and post hoc multiple comparison test against 0 condition (no E2 nor BPA). ∗ represents *p* < 0.05, ∗∗ for *p* < 0.01, and ∗∗∗ for *p* < 0.001.

We also determined the surface expression of MHC class II and costimulatory molecules as another measure of the response of cDCs to TLR stimulation and ability to activate. As with the proinflammatory cytokines, the upregulation of MHC class II and the costimulatory molecules CD40, CD86, and CD80 induced by CpG was completely estrogen-dependent and the higher levels of E2 did not promote any further increase in the upregulation of costimulatory molecules by CpG (Figures 4(b), 4(e), and 4(h) and Supplemental Figure 3B). Representative plots are shown in Supplemental Figure 4.

The response to LPS instead was only partially dependent on E2, since MHC class II, CD86, and CD40 were upregulated by LPS in the absence of E2, although MHC class II and CD86 expression was further increased in cDCs grown in E2 (Figures 4(c), 4(f), and 4(i)). CD80 reached a significant increase only in the presence of E2 (Supplemental Figure 3C). The higher concentration of E2 induced a similar increase in costimulatory molecules that did not reach statistical significance because of variability between experiments (Figures 4(c) and 4(f) and Supplemental Figure 3C).

The exposure of cDCs to the E2 analogue BPA did not significantly affect the expression of either MHC class II or costimulatory molecules (Figures 4(a), 4(b), 4(c), 4(d), 4(e), 4(f), 4(g), 4(h), and 4(i) and Supplemental Figure 3), indicating that BPA does not mimic the estrogen promotion of surface molecule expression required for the antigen-presenting functions of conventional DCs.

We have also noticed that, in the absence of any TLR stimulation (Figures 4(a), 4(d), and 4(g)), CD86 expression was slightly but significantly upregulated in cDCs grown in high E2, while CD40 and CD80 were very similar in all the unstimulated cDCs (Figures 4(d) and 4(g) and Supplemental Figure 3A). In addition, the expression of MHC class II was significantly increased by both low and high doses of E2 (Figure 4(a)). BPA did not have significant effects on these parameters (Figures 4(a), 4(d), and 4(g)). These results suggest that E2 not only improves the ability of cDCs to respond to TLR activation but also increases the constitutive expression of the MHC and costimulatory molecules, possibly preparing cDCs to induce tolerance [7], further supporting the role of estrogens in cDC differentiation. On the contrary, we did not find any evidence that BPA promotes any tested function of cDCs.

### 3.7. Estrogen but Not BPA Increases the Expression of TLR9

**Figure 5:**
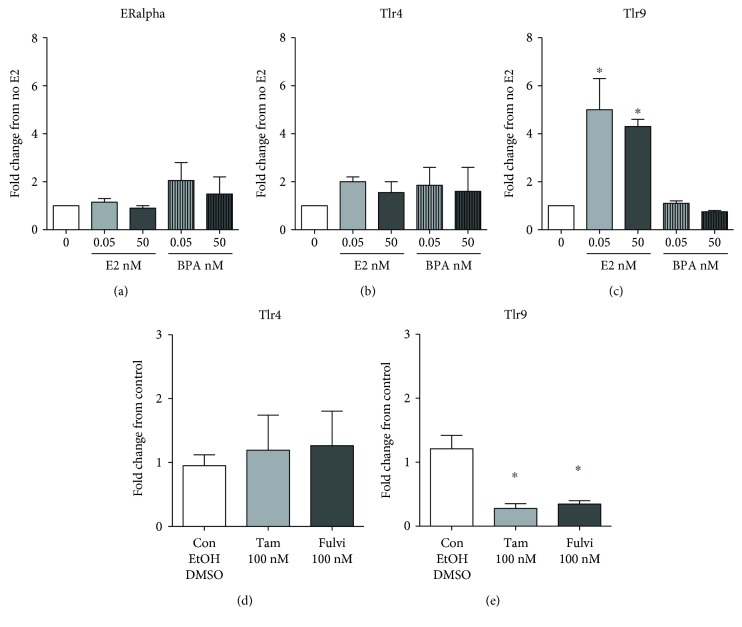
Estrogen but not BPA increases the gene expression of TLR9. We grew cDCs in the absence or presence of low (0.05 nM) and high doses (50 nM) of E2 or BPA for 6-7 days. Then we measured the total RNA expression of ERalpha1 (a), Tlr4 (b), and Tlr9 (c) by real-time quantitative RT-PCR. Results are averages and SE of two cDC cultures. (d) and (e) show the results of the real-time quantitative RT-PCR for TLR4 and TLR9 transcripts in cDCs grown in the presence of tamoxifen or fulvestrant. Results are averages and SE of three cDC cultures. We calculated statistical significance by one-way ANOVA and post hoc multiple comparison test against 0 condition (no E2 nor BPA) in (a–c) and against control in (d-e). Since the three controls medium alone, ethanol, and DMSO gave comparable results, we pooled the data. ∗ represents *p* < 0.05, ∗∗ for *p* < 0.01, and ∗∗∗ for *p* < 0.001.

We have so far presented results indicating that estrogen promotes the differentiation of cDCs and increases cDC response to TLR ligands, specifically augmenting their response to the TLR9 ligand CpG and, to a lesser extent, to the TLR4 ligand LPS. BPA has shown no effect. To understand the mechanisms of the E2 effects and lack of BPA effects on cDC differentiation and activation, we first measured the RNA expression of *ERalpha*, the main receptor that mediates the response to E2, and possibly to BPA, in cDCs [16]. We measured the expression of *ERalpha* RNA by real-time quantitative RT-PCR in cDCs generated in the absence or presence of E2 and BPA as shown in Figure 1(). We found that indeed cDCs, generated in our conditions, express ERalpha and neither E2 nor BPA modify such expression, suggesting that the effects of E2 on cDC activation are not modulated through the regulation of the receptor (Figure 5()). Then, we measured the expression of *Tlr4* and *Tlr9* RNA and found that E2 did not affect the expression of *Tlr4* (Figure 5(b)), but it induced a significant increase in the expression of *Tlr9* (Figure 5()C). This is suggestive of the ability of E2 to increase cDC activation at least partially by mediating the increase in TLR9 expression. Experiments in the presence of tamoxifen and fulvestrant confirmed these results, since they did not affect the expression of *Tlr4* while significantly decreasing the expression of *Tlr9* (Figures 5(d) and 5(e)). BPA did not induce any significant effect in TLR expression, in line with its lack of effects on cDC activation (Figures 5(b) and 5(c)).

## 4. Discussion

The investigation of the effects of estrogens and xenoestrogenic pollutants on the immune system, and on DCs particularly, is an exciting but still controversial field due to the conflicting results reported in the literature [16, 19]. Many papers have suggested proinflammatory functions for estrogens because their depletion in vitro reduced DC differentiation and DC response to TLR ligands [31]. However, a few papers have reported anti-inflammatory effects of estrogens, such as the inhibition of NF-kB activation [48] and the induction of IL-10 [49]. We have used a protocol allowing the generation of cDCs in the absence of estrogens. This was possible due to IMDM, a highly enriched synthetic medium, originally developed for culturing cells in serum-free conditions [50] and for highly demanding cultures. IMDM may provide enough nutrients to overcome the absence of sex hormones. Using this tool, we have extended the evidence that estrogen enhances cDC differentiation because we found that estrogen increased the percentages of cDCs. Moreover, our results support previous evidence of a proinflammatory effect of estrogen on DC physiology [31, 34, 35, 51]. We show that conventional DCs grown in hormone-depleted medium are impaired in their response to TLR stimulation, with a major impact on the response to the TLR9 ligand CpG. The generation of cDCs in a complete medium, supplemented with a standard FBS and in the presence of the SERMs tamoxifen and fulvestrant, provides a complementary approach to confirm the role of estrogen in cDC differentiation and activation and excludes other steroidal hormones or lipidic compounds that are eliminated by the treatment of FBS with charcoal.

BPA is pervasive in our environment and has been recognized as a toxic compound that should be eliminated from infant bottles and other food containers. Unfortunately, laws to implement these requirements are in place in only a few industrialized countries [19]. Furthermore, BPA-containing products disposed of in garbage fields can leach and BPA continues to infiltrate the water supply [20–27]. The constant exposure of the population to BPA (over 90% of US citizens have detectable urinary levels of BPA [43]) exemplifies a continued need to understand the physiological effects of this endocrine-disrupting chemical. We tested the estrogenic-like potential of BPA on cDCs and found that BPA did not mimic the estrogenic functions because it did not affect cDC differentiation nor their ability to respond to TLR stimulation. Our results are in agreement with more recent reports showing a lack of effect of BPA on immune cells in vivo and on disease development in inflammatory colitis and anti-influenza host defense [39, 40].

We have analyzed the role of estrogen and BPA in an in vitro model of cDCs that has been used in several papers and have highlighted the role of estrogen in their growth, differentiation, and activation and the lack of such effects by BPA. In order to clearly determine the role of estrogen on DC differentiation and function as a single variable, we have chosen to use a reductionist approach and implement an in vitro model, in which only the cell subset of interest is present. Our results warn to consider that different effects of lots of FBS on cDCs can be at least in part explained by differences in the concentration of estrogens.

Estrogen forms a complex with the estrogen receptor, which acts as a transcription factor and can directly regulate gene expression by binding to estrogen response elements (ERE) in estrogen-dependent genes. We have found putative EREs within the genes and surrounding regions of CD11c and CD11b, suggesting that one possible mechanism for the stimulating effects of estrogens on cDC differentiation could involve direct enhancement of gene expression (Supplemental Figure 5). Similarly, we found several EREs in the sequences of IL-12 and TNF*α* and the costimulatory molecules affected by estrogens. This direct binding of estrogens to EREs cannot explain why E2 modulates cDC response to CpG and, to a lesser extent, LPS. Such differential modulation can be explained instead by the increase in the *Tlr9* expression that we found induced by E2. Therefore, estrogens can affect cDC response to CpG by directly affecting the production of cytokines and costimulatory molecules and the expression of *Tlr9*. Moreover, estrogens may affect nonclassical signaling pathways downstream of TLRs such as the phosphorylation of pivotal kinases [19, 52].

In our results, estrogen had similar effects on cDCs at diestrus levels and at higher levels (0.05 nM versus 50 nM). This is surprising because previous work has found a dose-dependent response of DCs to estrogen, but technical differences in the protocol to generate and activate DCs can explain the dissimilarity in sensitivity to different concentrations of estrogen.

It has been suggested that estrogens increase the general expression of costimulatory molecules in cDCs and pDCs upon LPS and CpG stimulation [34, 53]. Our results specify that the costimulatory molecules CD86, CD80, and MHC class II are upregulated upon LPS and CpG stimulation mostly in the presence of E2, while LPS-induced CD40 upregulation is more estrogen-independent, confirming previous data [29] that CD86 and MHC class II upregulation was absent in ERalpha^−/−^ DCs, while CD40 upregulation was normal. The induction of IL-12 and TNF*α* in ERalpha^−/−^ DCs was increased upon LPS stimulation while it was reduced with CpG [29], mirroring our results that IL-12 and TNF*α* are induced by LPS in the absence of estrogen, while all the responses to CpG are enhanced by estrogen. These results lead us to speculate that estrogen may be less important for the clearance of bacterial infections, driven by LPS-induced responses, while it may affect more TLR9-driven responses occurring during viral infections and autoimmunity. Indeed, in the autoimmune disease systemic lupus erythematosus, TLR9 is an important molecular mediator of stimulation of the innate and adaptive immune responses that drive the autoimmune process [54]. TLR9 is triggered by the main autoantigen in lupus, double-stranded DNA (dsDNA), and by the immune complexes carrying DNA and leads to DC hyperactivation and stimulation of autoreactive B cells [14]. Our results show that estrogen enhances DC responses to TLR9 ligand CpG which suggests that estrogen could fuel the innate response to the main auto-antigens in lupus and amplify the production of the autoantibodies that would complete the vicious circle.

We also found that BPA did not increase such cDC response to TLR9. BPA has been proposed as an immune stimulator, but evidence is lacking for a direct role in lupus pathogenesis. Moreover, it has been reported that treatment of lupus-prone mice in vivo with BPA led to delayed autoimmunity with a reduced production of cytokines by T cells and autoantibodies by B cells, suggesting anti-inflammatory effects of BPA on T and B lymphocytes [55]. Our results neither support nor disprove these observations, but rather suggest that the in vivo effects of BPA on the immune response and pathogenesis of autoimmune diseases reported in the literature may require synergisms with other immune modulators or they ought to derive from BPA effects on cells other than cDCs [19, 36–39]. We suggest the need of further investigation to better understand the effects of BPA on the innate and adaptive immune response.

## Supplementary Material

Supplemental Figure 1. Estrogen but not BPA protects cDCs from LPS-induced cell death. We grew cDCs with and without E2 or BPA as described in Figure 1 and then stimulated cDCs at day 6-7 with 100 ng/ml of LPS and then performed manual counts of live cells by excluding cells positive for the trypan blue staining. Results are shown as percentages of live cells for each cDC sample grown in E2, BPA or in absence of E2 and stimulated by LPS for 24 hours, and compared to the unstimulated cDCs normalized to 100% for each culture condition. The results are averages and SE of four experiments conducted with four independent BMDC cultures. Statistical differences were calculated against No E2. Supplemental Figure 2. Estrogens neither enhance nor inhibit the development of inflammatory CD11b+Ly6C+Ly6G+ subset of innate immune cells. (A-B) Percentage of cells that stained positive for anti-Gr1 Ab, which recognizes both Ly6C+ and Ly6G+ cDay 6-7 of culture of cDCs grown in hormone-depleted conditions in the absence or presence of lowells, and anti-CD11b. (A) (0.1 nM) and high doses (50 nM) of 17 beta-estradiol (E2). (B) Day 6-7 of culture of cDCs grown in the regular medium and in the absence or presence of 1μM Fulvestrant or 100 nM Tamoxifen: results are averages and SE of biological duplicates conducted with two to 3 independent cDC cultures. Statistical significance was calculated by one-way ANOVA and post-hoc multiple comparison test against the 0 condition (No E2) in A and against Control in B. Throughout all the figures of this article, ∗ is for p < 0.05; ∗∗ for p < 0.01, and ∗∗∗ for p< 0.001. Supplemental Figure 3. Estrogen but not BPA up-regulates the expression of the costimulatory molecule CD80 upon stimulation with CpG and LPS. As in Figure 3, the percentage of cDCs (gated for CD11c+) positive for the costimulatory molecule CD80 is shown in absence of stimulation (A) or after 24 hours of stimulation with CpG (B) or LPS (C). We calculated statistical differences with the cDCs generated without E2. Supplemental Figure 4. Estrogen but not BPA up-regulates the expression of the costimulatory molecule CD86 upon stimulation with CpG and LPS. (A-I) Plots showing the expression of the cDC differentiation marker CD11c versus the activation marker CD86 as analyzed by the Flow Jo software. The small numbers in the quadrants represent the percentages of cells positive for the indicated markers in the total alive population. The bigger numbers in the upper right corner represent the percentage of CD86 positive cells in the CD11c+ population, and are shown in Fig. 3. Supplemental Figure 5. Putative Estrogen Response Elements (ERE) within the genes and the surrounding regions of immunologically relevant molecules. Putative estrogen response elements found in genes (green) and in 10kB regions surrounding the genes (yellow) of C57BL/6 mice (surrounding region not shown for MHC II). Sequences were obtained from the Mouse Genome Informatics Website and searched for known ERE using Dragon ERE Finder version 6. Red lines indicate ERE in forward direction, blue lines indicate ERE in reverse direction. Numbers in parenthesis are kilobases. All searched genes contain putative ERE.

## Abbreviations

APC: Allophycocyanin
BM: Bone marrow
BPA: Bisphenol A
cDC: Conventional dendritic cell
CpG: CpG oligonucleotide sequences
E2: Beta-estradiol 3-benzoate, 17beta-estradiol
ER: Estrogen receptor
FBS: Fetal bovine serum
FITC: Fluorescein isothiocyanate
TLR: Toll-like receptor
LPS: Lipopolysaccharide
IL: Interleukin
TNF*α*: Tumor necrosis factor a
pDCs: Plasmacytoid dendritic cells
IMDM: Iscove's Modified Dulbecco Medium
RPMI: Roswell Park Memorial Institute medium
PRFCT IF-10: Phenol red-free IMDM supplemented with 10% charcoal-treated heat-inactivated fetal bovine serum
PRFCT R-10: Phenol red-free RPMI supplemented with 10% charcoal-treated heat-inactivated fetal bovine serum
GM-CSF: Granulocyte macrophage colony-stimulating factor
PE: Phycoerythrin
MHC: Major histocompatibility complex.
