# Supplementary material for: Bisphenol A Does Not Mimic Estrogen in the Promotion of the In Vitro Response of Murine Dendritic Cells to Toll-Like Receptor Ligands
Source: Mediators Inflamm. 2017 Jul 25;2017:2034348. doi: 10.1155/2017/2034348 (PMC5547709; doi:10.1155/2017/2034348)
Supplement: Supplementary file 1 — Supplemental Figure 1. Estrogen but not BPA protects cDCs from LPS-induced cell death. We grew cDCs with and without E2 or BPA as described in Figure 1 and then stimulated cDCs at day 6-7 with 100 ng/ml of LPS and then performed manual counts of live cells by excluding cells positive for the trypan blue staining. Results are shown as percentages of live cells for each cDC sample grown in E2, BPA or in absence of E2 and stimulated by LPS for 24 hours, and compared to the unstimulated cDCs normalized to 100% for each culture condition. The results are averages and SE of four experiments conducted with four independent BMDC cultures. Statistical differences were calculated against No E2. Supplemental Figure 2. Estrogens neither enhance nor inhibit the development of inflammatory CD11b+Ly6C+Ly6G+ subset of innate immune cells. (A-B) Percentage of cells that stained positive for anti-Gr1 Ab, which recognizes both Ly6C+ and Ly6G+ cDay 6-7 of culture of cDCs grown in hormone-depleted conditions in the absence or presence of lowells, and anti-CD11b. (A) (0.1 nM) and high doses (50 nM) of 17 beta-estradiol (E2). (B) Day 6-7 of culture of cDCs grown in the regular medium and in the absence or presence of 1μM Fulvestrant or 100 nM Tamoxifen: results are averages and SE of biological duplicates conducted with two to 3 independent cDC cultures. Statistical significance was calculated by one-way ANOVA and post-hoc multiple comparison test against the 0 condition (No E2) in A and against Control in B. Throughout all the figures of this article, ∗ is for p < 0.05; ∗∗ for p < 0.01, and ∗∗∗ for p< 0.001. Supplemental Figure 3. Estrogen but not BPA up-regulates the expression of the costimulatory molecule CD80 upon stimulation with CpG and LPS. As in Figure 3, the percentage of cDCs (gated for CD11c+) positive for the costimulatory molecule CD80 is shown in absence of stimulation (A) or after 24 hours of stimulation with CpG (B) or LPS (C). We calculated statistical differences with t [file 2034348.f1.docx]

Supplemental Figure 1:


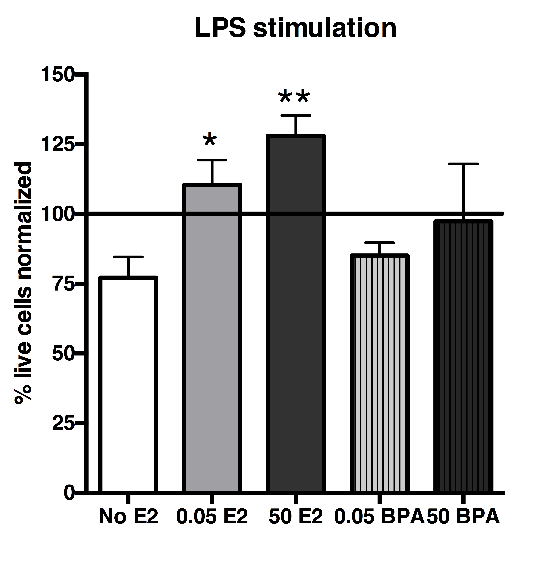


*Supplemental Figure 1. Estrogen but not BPA protects cDCs from LPS-induced cell death.* We grew cDCs with and without E2 or BPA as described in Figure 1 and then stimulated cDCs at day 6-7 with 100 ng/ml of LPS and then performed manual counts of live cells by excluding cells positive for the trypan blue staining. Results are shown as percentages of live cells for each cDC sample grown in E2, BPA or in absence of E2 and stimulated by LPS for 24 hours, and compared to the unstimulated cDCs normalized to 100% for each culture condition. The results are averages and SE of four experiments conducted with four independent BMDC cultures. Statistical differences were calculated against No E2.

Supplemental Figure 2:


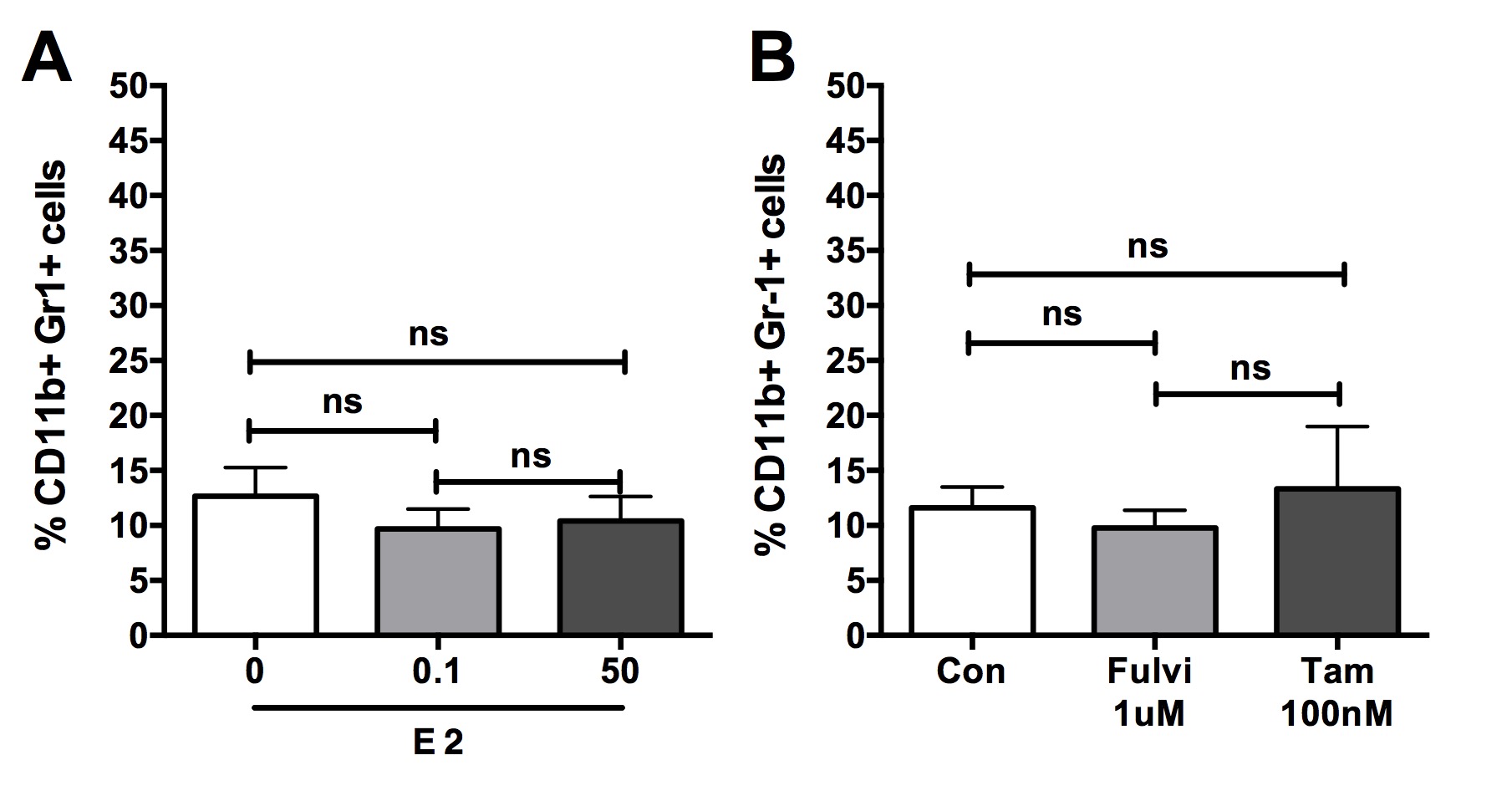


*Supplemental Figure 2. Estrogens neither enhance nor inhibit the development of inflammatory CD11b+Ly6C+Ly6G+ subset of innate immune cells.*  (A-B) Percentage of cells that stained positive for anti-Gr1 Ab, which recognizes both Ly6C+ and Ly6G+ cells, and anti-CD11b. (A) Day 6-7 of culture of cDCs grown in hormone-depleted conditions in the absence or presence of low (0.1 nM) and high doses (50 nM) of 17 beta-estradiol (E2). (B) Day 6-7 of culture of cDCs grown in the regular medium and in the absence or presence of 1uM Fulvestrant or 100 nM Tamoxifen: results are averages and SE of biological duplicates conducted with two to 3 independent cDC cultures. Statistical significance was calculated by one-way ANOVA and post-hoc multiple comparison test against the 0 condition (No E2) in A and against Control in B. Throughout all the figures of this article, * is for *p* < 0.05; ** for *p <* 0.01, and *** for *p<* 0.001.

Supplemental Figure 3

A B C


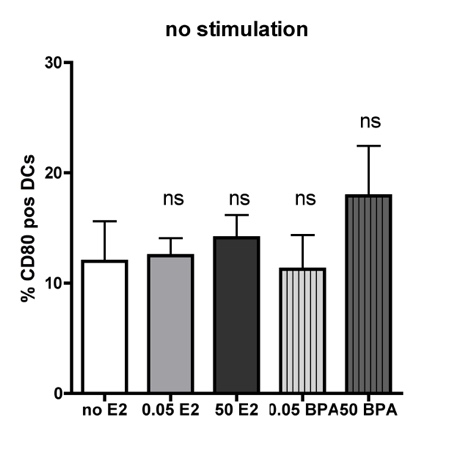

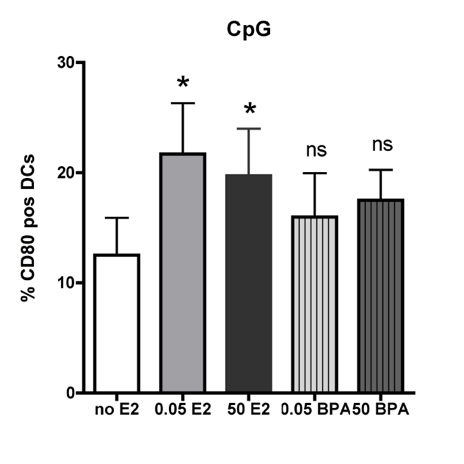

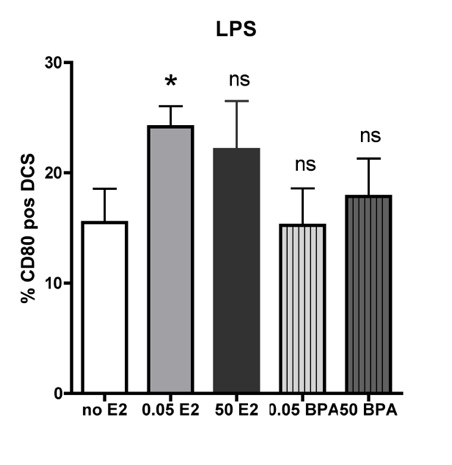


*Supplemental Figure 3. Estrogen but not BPA up-regulates the expression of the costimulatory molecule CD80 upon stimulation with CpG and LPS.* As in Figure 3, the percentage of cDCs (gated for CD11c+) positive for the costimulatory molecule CD80 is shown in absence of stimulation (A) or after 24 hours of stimulation with CpG (B) or LPS (C). We calculated statistical differences with the cDCs generated without E2.

Supplemental Figure 4:


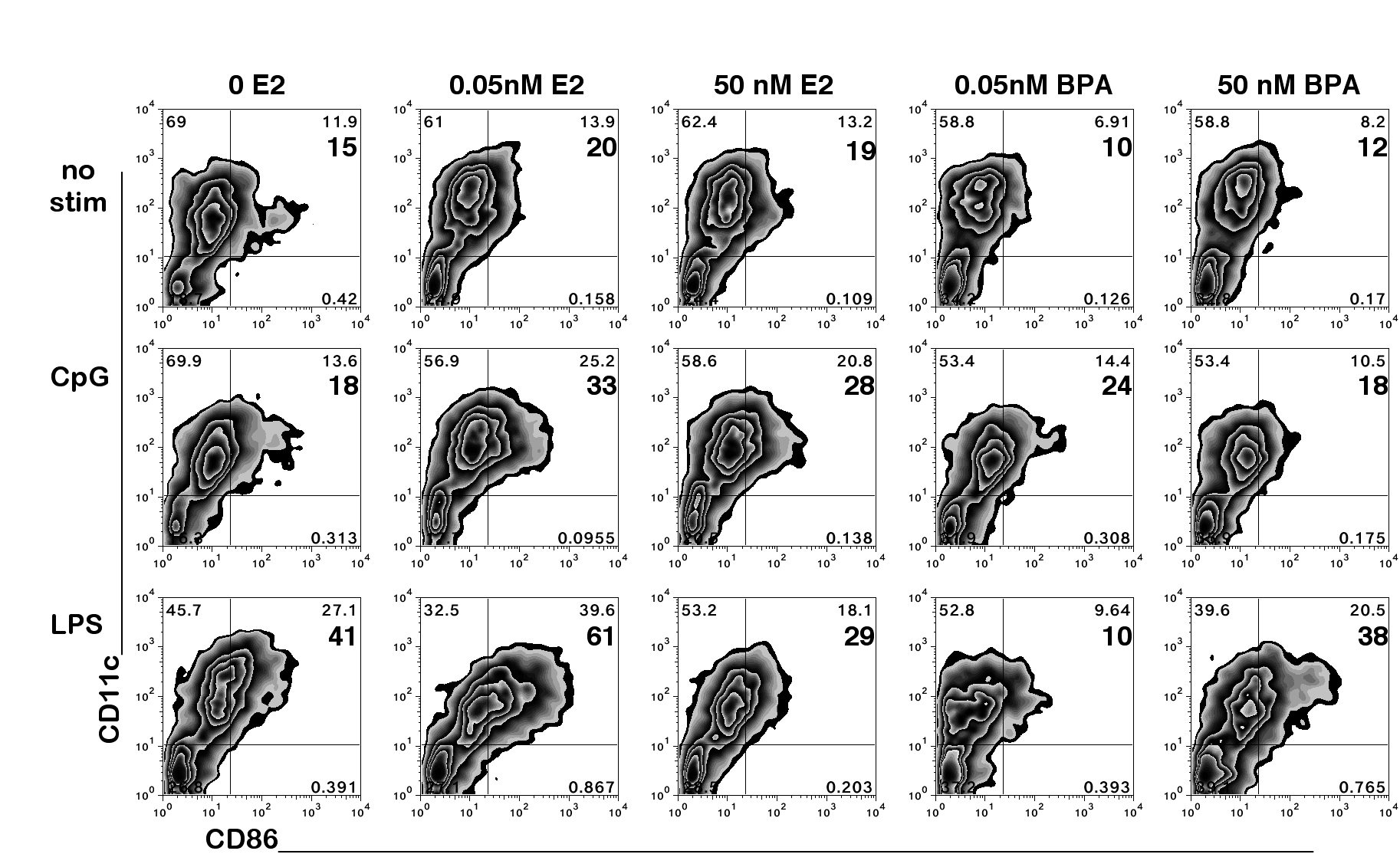


*Supplemental Figure 4. Estrogen but not BPA up-regulates the expression of the costimulatory molecule CD86 upon stimulation with CpG and LPS.* (A-I) Plots showing the expression of the cDC differentiation marker CD11c versus the activation marker CD86 as analyzed by the Flow Jo software. The small numbers in the quadrants represent the percentages of cells positive for the indicated markers in the total alive population. The bigger numbers in the upper right corner represent the percentage of CD86 positive cells in the CD11c+ population, and are shown in Fig. 3.

Supplemental Figure 5


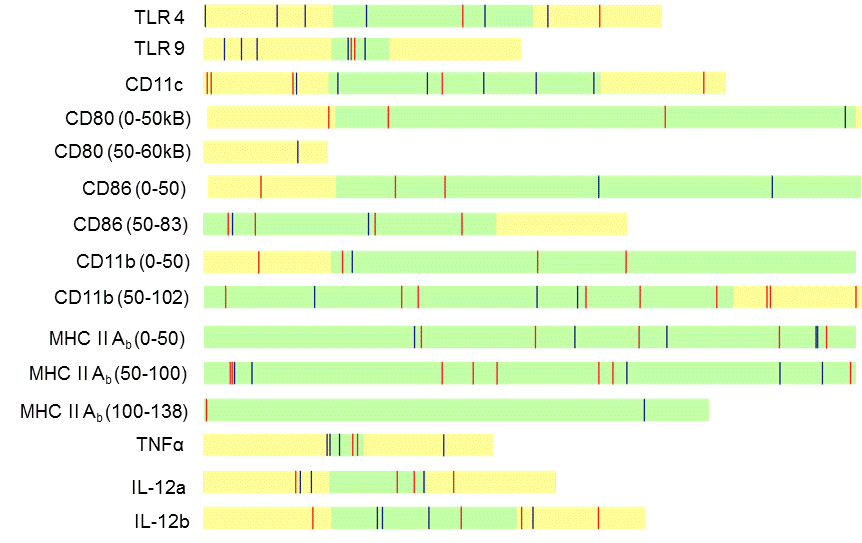


*Supplemental Figure 5. Putative Estrogen Response Elements (ERE) within the genes and the surrounding regions of immunologically relevant molecules.* Putative estrogen response elements found in genes (green) and in 10kB regions surrounding the genes (yellow) of C57BL/6 mice (surrounding region not shown for MHC II). Sequences were obtained from the Mouse Genome Informatics Website and searched for known ERE using Dragon ERE Finder version 6. Red lines indicate ERE in forward direction, blue lines indicate ERE in reverse direction. Numbers in parenthesis are kilobases. All searched genes contain putative ERE.
