# Supplementary figures and images for: Bisphenol A Does Not Mimic Estrogen in the Promotion of the In Vitro Response of Murine Dendritic Cells to Toll-Like Receptor Ligands
Source: Mediators Inflamm. 2017 Jul 25;2017:2034348. doi: 10.1155/2017/2034348 (PMC5547709; doi:10.1155/2017/2034348)

## LPS stimulation

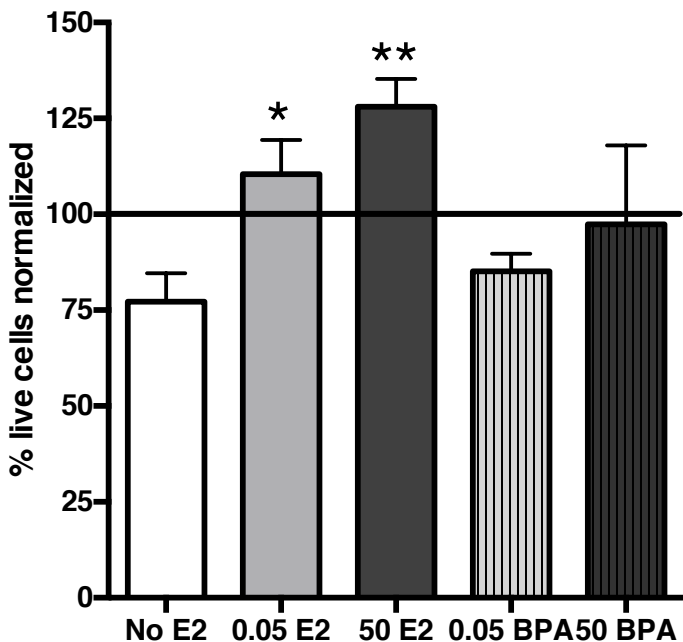

Supplement: Supplementary file 2 [file 2034348.f2.pdf]

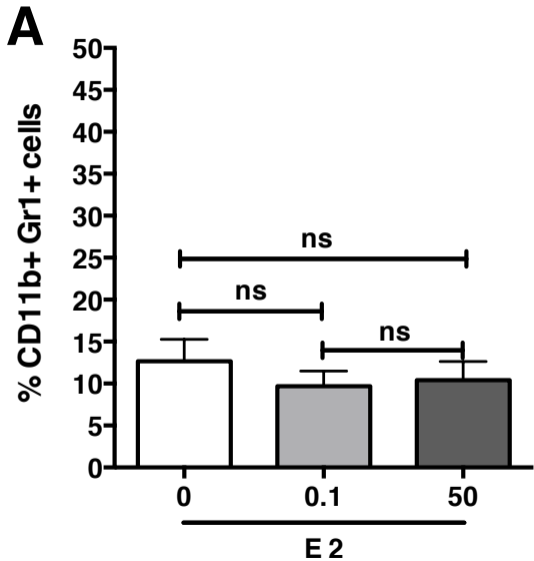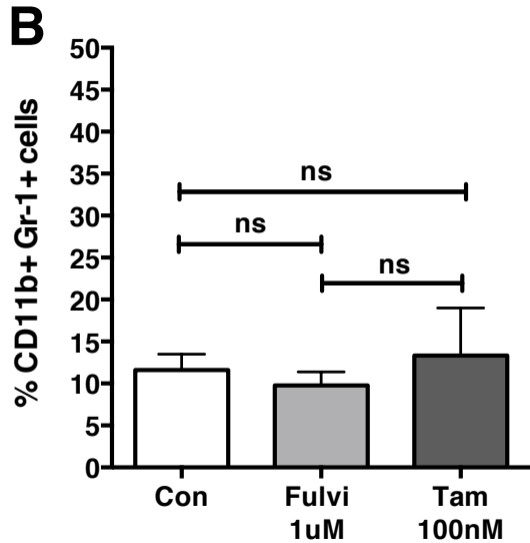

Supplement: Supplementary file 3 [file 2034348.f3.pdf]

no stimulation

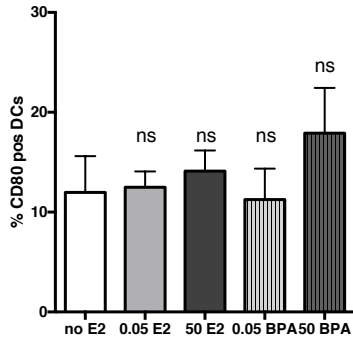

CpG

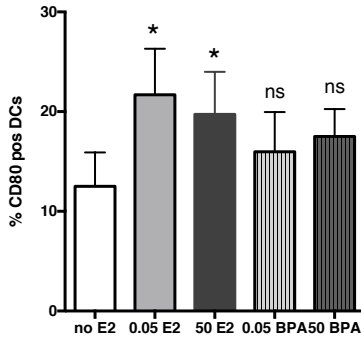

LPS

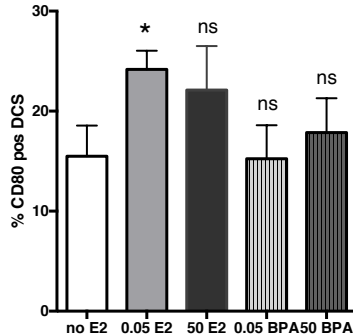

Supplement: Supplementary file 4 [file 2034348.f4.pdf]

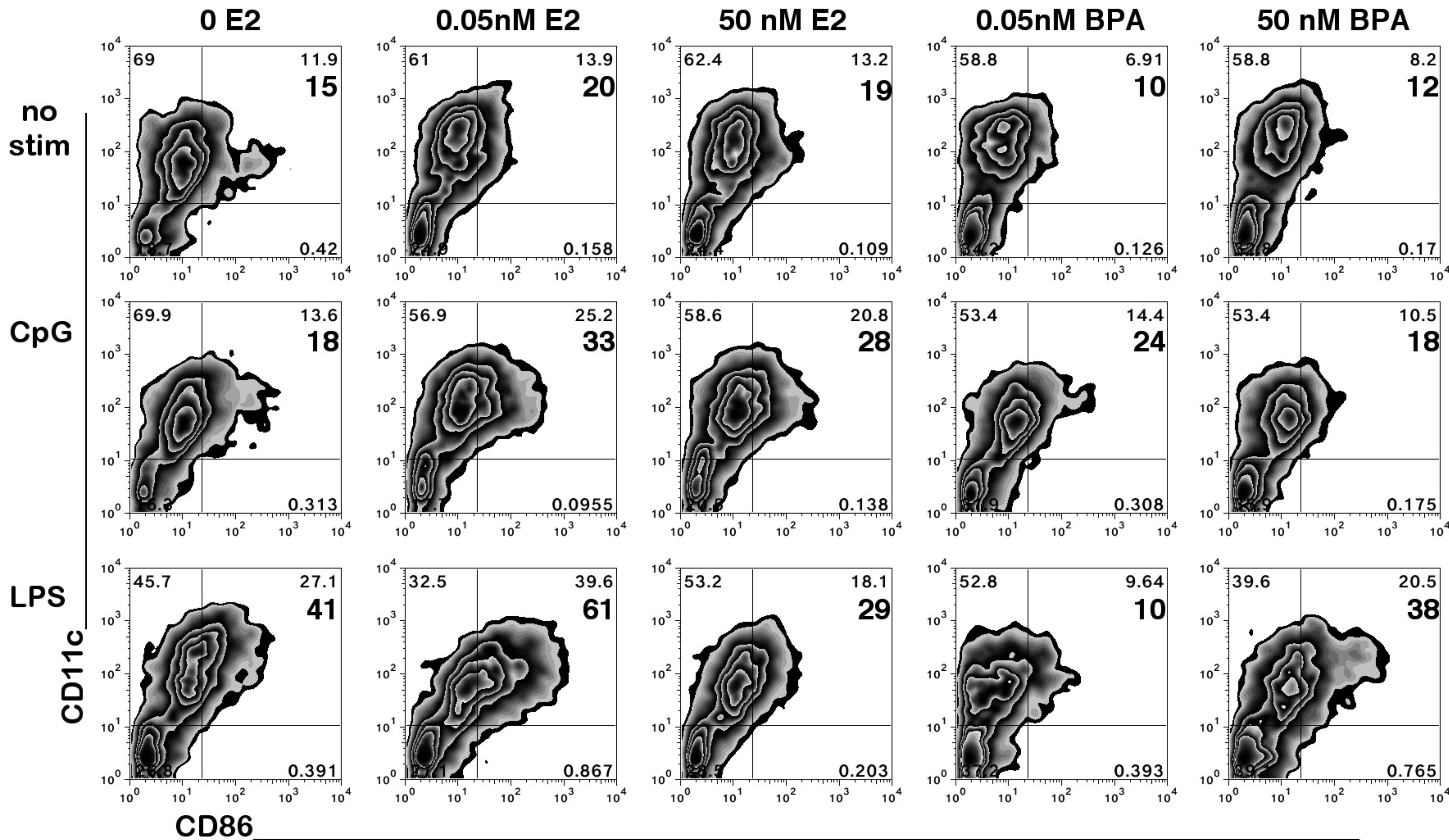

Supplement: Supplementary file 5 [file 2034348.f5.pdf]

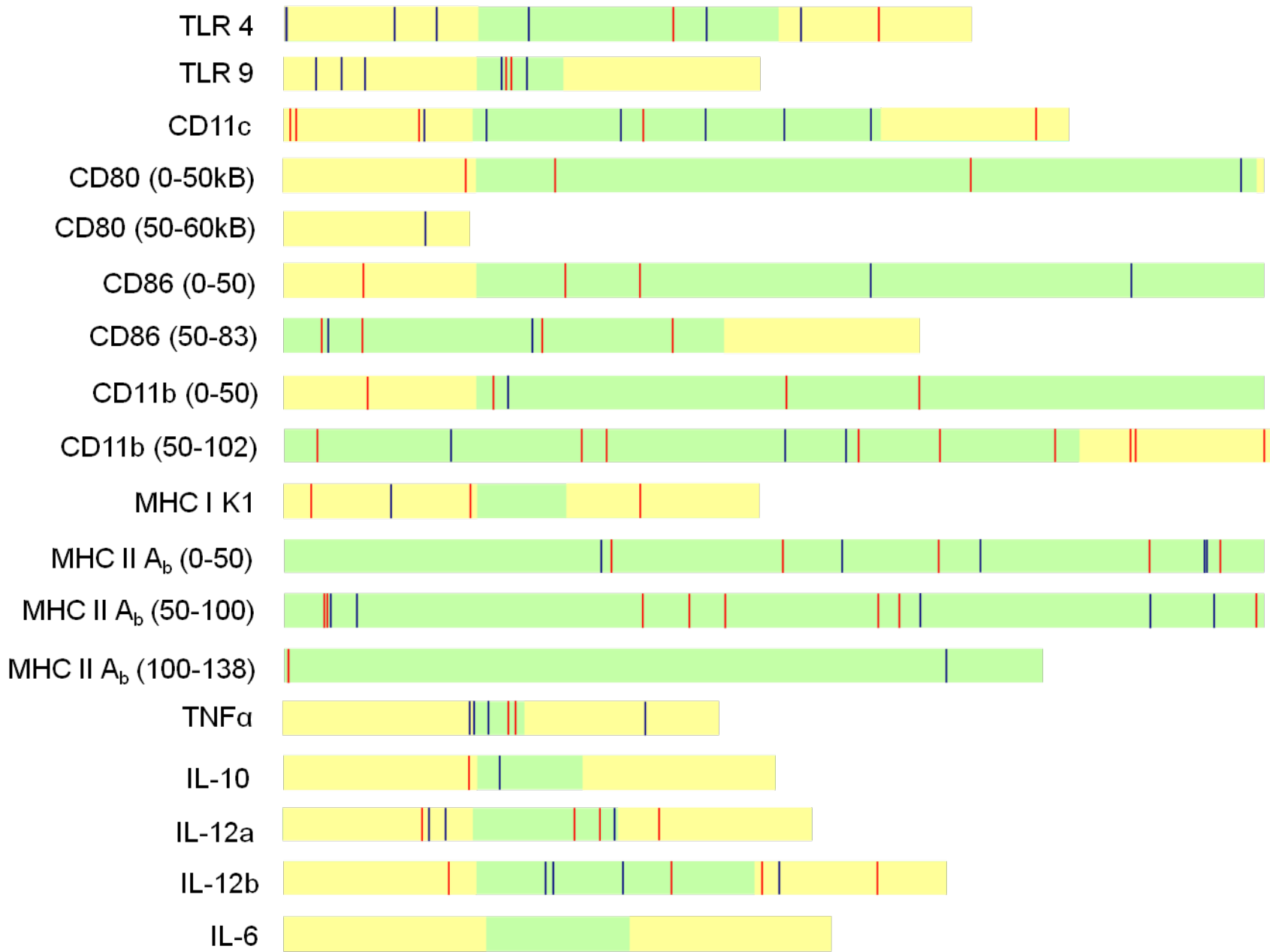

Supplement: Supplementary file 6 [file 2034348.f6.pdf]
